# Pharmacological basis for the medicinal use of polyherbal formulation and its ingredients in cardiovascular disorders using rodents

**DOI:** 10.1186/s12906-017-1644-0

**Published:** 2017-03-07

**Authors:** Abdul Malik, Malik Hassan Mehmood, Hajra Channa, Muhammad Shoaib Akhtar, Anwarul-Hassan Gilani

**Affiliations:** 1 Natural Product Research Division, Department of Biological and Biomedical Sciences, Faculty of Health Sciences, Medical College, The Aga Khan University, Stadium Road, P.O. Box 3500, Karachi, 74800 Pakistan; 2 Faculty of Pharmacy, University of Sargodha, Sargodha, 40100 Pakistan; 3 The Aga Khan University Medical College, Stadium Road, P.O. Box 3500, Karachi, 74800 Pakistan; 4 Pakistan Council for Science and Technology, G-5/2, Islamabad, 44000 Pakistan

**Keywords:** POL_4_, Antihypertensive, Vasomodulatory, Ca^++^ antagonist, α-adrenergic antagonist

## Abstract

**Background:**

A compound herbal formulation (POL_4_) has been used in the indigenous system of medicine to treat cardiometabolic disorders like diabetes and associated hypertension. POL_4_ and most of its constituents have not been studied widely for its therapeutic use in hypertension. This study is aimed to determine the efficacy and possible insight into mechanism(s) for the medicinal use of POL_4_ and its ingredients in hypertension.

**Methods:**

The aqueous methanolic extracts of POL_4_ (POL_4_.Cr) and its components [*Cichorium intybus* (Ci.Cr), *Gymnema sylvestre* (Gs.Cr), *Nigella sativa* (Ns.Cr) and *Trigonella foenum graecum* (Tfg.Cr)] were tested for blood pressure lowering activity in anaesthetized Sprague-Dawley rats. To assess the vasomodulatory effect, isolated tissue experiments were performed on rat aortic strips using isometric force transducer coupled with PowerLab data acquisition system.

**Results:**

Administration of POL_4_ to rats caused a dose-dependent (1–100 mg/kg) fall in mean arterial pressure (MAP) with maximum effect of 85.33 ± 1.76% at 100 mg/kg, similar to the effect of verapamil. All ingredients of POL_4_ also decreased blood pressure with varying efficacy in following order Ns.Cr ≅ Ci.Cr > Tfg.Cr > Gs.Cr. In rat aortic preparations, POL_4_ and its ingredients inhibited K^+^ (80 mM)-induced contractions, Ci.Cr was the most potent followed by Ns.Cr > Tfg.Cr > Gs.Cr ≅ POL_4_. Against phenylephrine (P.E) contractions, Ci.Cr and Tfg.Cr exhibited complete relaxation, while POL_4_.Cr, Gs.Cr and Ns.Cr showed vasomodulatory effect. The Ca^++^ antagonist activity was confirmed when POL_4_ and its ingredients shifted Ca^++^ concentrations-response curves to the right in a manner similar to that of verapamil. On baseline of rat aorta, the parent formulation and its ingredients (except Tfg.Cr) exhibited partially phentolamine (1 μM)-sensitive vasoconstriction.

**Conclusion:**

These data show that POL_4_ and its constituents possess blood pressure lowering activity mediated through inhibition of Ca^++^ influx via membranous Ca^++^ channels and receptor (α-adrenergic) operated pathways. Thus, this study provides a rationale to the medicinal use of POL_4_ and its constituents in hypertension.

## Background

Cardiovascular disorders (CVDs) are huge disease burden worldwide [1]. Hypertension, dyslipidemia and endothelial dysfunctions are the major pathologies of commonly prevailing CVDs [2]. Hypertension is a silent killer, which is known to cause around 9.8 million deaths annually [3]. Pakistan is one of the South Asian countries where around 18 million people at the age of fifteen and above are living with hypertension. Out of these, around 70% population is even unaware about the disease status, while only 3% successfully control blood pressure with regular medication [4–6]. Multiple antihypertensive therapeutic agents are available to combat this deadly disorder, however, their high cost and life-long use of the medication along with associated multiple adverse effects ultimately results in patient unaffordability and poor-compliance [7]. The current strategy to defeat chronic non-communicable disorders is changing throughout the world. The use of alternative approaches, particularly medicinal remedies and supplements [8, 9], have become popular over the past three decades. Around 80% of people worldwide rely on natural products [10] for therapeutic purpose. Treating chronic disorders like hypertension or diabetes, the herbal remedies are considered relatively safe, affordable and easily accessible. The relative safety of natural products when used for longer duration might be because of the presence of synergistic and/or side effects neutralizing constituents inbuilt in medicinal herbs [11]. In our settings due to cultural acceptance, people prefer to use herbal remedies for the treatment of various chronic ailments like gastro-intestinal [12], respiratory tract [13], endocrine (diabetes) [14] and cardiovascular disorders [15]. Despite using individual herbs to treat different ailments, there are multiple evidences supporting therapeutic use of compound formulations in the treatment of hypertension and dyslipidemia. Some of the combined formulations like Sharbat Abresham, Hab Fishar for hypertension, Arq-Sheer-Morakkab as cardiotonic, Muffrahe-Barid-Sada and Arq-Gazar Ambari as anti-palpitant [16] are used in indigenous system of medicine in Pakistan. POL_4_, an indigenous polyherbal formulation containing four edible medicinal herbs (*Cichorium intybus* L*.*, *Gymnema sylvestre* R. Br.*, Nigella sativa* L*.,* and *Trigonella foenum graecum* L*.*) is also popular for its medicinal use in cardiometabolic disorders, however, no study is available to rationalize its medical use in cardiometabolic disorders like hypertension.

The ingredients of POL_4_: *C. intybus* (family: Asteraceae) commonly called Chicory or Kasni, *G. sylvestre* (family: Asclepiadaceae) known as Gurmar booti, *N. sativa* (family: Ranunculaceae) referred as Black cumin or Kalonji and *T. foenum graecum* (family: Fabaceae) vernacularly called as Fenugreek or Methi are popular for their medicinal use as cardiotonic, antihypertensive and cadio-depressant [17]. *C. intybus* (coumarins, sterols, tannins, triterpene cichoridiol, lupeol, sesquiterpene glycoside, caffeic acid and cichotyboside) [18], *G. sylvestre* (gymnemasides anthraquinone, stigmasterol, betaine, quercitol and gymnemagenin) [19], *N. sativa* (thymoquinone, α-pinene, p-cymnene, carvacrol, terpineol, nigellicimine and nigellidine) [20] and *T. foenum graecum* (trigonelline, cholin, gentian, carpaine and 4-hydroxyisoleucine and diosgenin) [21] have been found enriched with variety of phytochemicals. Most of the determined phytochemicals on the part of the components of POL_4_ are also known for their diverse health benefits including their effectiveness in cardiovascular disorders [22–27]. Though detailed studies are lacking to justify the cardiovascular benefits of POL_4_ and its ingredients, the available data is providing some support to individual components {*C. intybus* [27, 28], *G. sylvestre* [19, 29] and *T. foenum graecum* [30, 31]} for their utility in cardiovascular disorders with exception of *N. sativa* which has been studied widely. Despite the availability of ample data on *N. sativa* and its constituents (thymoquinone, α-pinene and p-cymene) for their effectiveness in hypertension [24–26, 32–34], the effect of its seed extract with holistic picture on vascular and atrial preparations is yet to be determined for its modulating role on vascular resistance and cardiac output. This study has been designed to provide an evidence to the medicinal use of POL_4_ and its ingredients in cardiovascular disorders like hypertension with a prime objective to determine the blood pressure lowering effects and the possible insight into mechanism(s). While the secondary objective was to compare the efficacy of ingredients with the parent formulation to justify the use of this formulation in hypertension and/or to suggest modification in its current composition. The in-vivo assays were carried out in rat as per their similarity with human cardiovascular system, which are commonly employed worldwide to determine antihypertensive efficacy of the test compounds [35–37], while isolated tissue experiments were conducted in rat (aorta) and guinea-pig (atria) tissues.

## Methods

### Preparation of aqueous-methodic extracts of POL_4_ and its ingredients

The plant materials were purchased from “Rehmania Pinsar Store” Sargodha and the samples were identified by Dr. Muhammad Amin Ullah Shah, Assistant Professor of Botany, Department of Botany, University of Sargodha, Sargodha, Pakistan. The respective samples have been submitted in the herbarium at University of Sargodha with voucher numbers as: [seeds of *N. sativa* (Malik-632), *C. intybus* (Malik-633)*, T. foenum graecum* (Malik-634) and leaves of *G. sylvestre* (Malik-635)]. The individual plant materials and in combination (POL_4_) were subjected for extraction. For preparation of the crude extract, 1 kg of powder of each plant material and 1 kg of POL_4_ (all ingredients in equal proportions) were soaked, respectively, in aqueous methanol (30: 70) for 3 days with occasional shaking. The methanol of analytical grade was purchased from Merck KGaA, Darmstadt, Germany. The first filtrate obtained was subjected to filter using muslin cloth and Whatman (Maidstone, UK) No.1 filter paper simultaneously. The procedure was repeated twice and all filtrates were combined and evaporated under rotary evaporator (BUCHI, Switzerland) [38]. The filtrates were then air dried and the respective percentage yields were calculated for the crude extracts of polyherbal formulation POL_4_.Cr (16% w/w), *C. intybus, Ci.Cr* (11% w/w), *G. sylvestre*, *Gs.Cr* (18% w/w), *N. sativa*, *Ns.Cr* (12% w/w) and *T. foenum graecum*, Tfg.Cr (17.5% w/w).

### Drugs and chemicals

Verapamil hydrochloride, potassium chloride, acetylcholine chloride, phenylephrine, phentolamine, norepinephrine, isoproterenol and calcium chloride were purchased from Sigma Chemical Company, St. Louis, MO, U.S.A. Glucose, sodium chloride, magnesium sulphate, magnesium chloride, potassium dihydrogen phosphate, sodium bicarbonate and sodium dihydrogen phosphate were from E. Merck, Darmstadt, Germany. All chemicals used were of the highest purity grade. Physiological salt solutions were prepared fresh in distilled water on the day of experiment, while stock solutions of all chemicals and extracts were constituted in distilled water/saline and stored at −20 °C, while required dilutions were prepared fresh on the day of the experiment.

### Animals, diet and experimental protocol

The Sprague-Dawley (SD) rats weighing 180–250 g and locally bred guinea pigs weighing 450–550 g healthy of either sex housed at the animal house of Aga Khan University, Karachi, were used in this study. Animals were either sourced from PCSIR (Pakistan Council of Scientific and Industrial Research, Karachi or Dow University of Health Sciences Karachi and have also been managed to be bred at the animal house of Aga Khan University. The animals were housed in a temperature maintained facility (23 ± 5 °C, 55 ± 5%, relative humidity) with 12-hr light/dark cycle, kept in plastic cages with sawdust (renewed at every two days). Animals had free access to standard diet [(g/kg): flour 380, fiber 380, molasses 12, sodium chloride 5.8, nutrivet-L 2.5, potassium metabisulphate 1.2, vegetable oil 38, fish meal 170 and powdered milk 150] and tap water, and were acclimatized for 5 to 7 days before starting experiment. The experimental protocols were approved (57-ECACU-BBS-15) by Ethics Committee for Animal Care and Use (ECACU) of the Aga Khan University, Karachi.

### Phytochemical screening

Phytochemical screening of the crude extract of POL_4_ and its constituents were carried out for the presence of flavonoids, tannins, saponins, alkaloids, anthraquinones and coumarins by using an earlier described method with slight modification [39].

### Determination of total phenolic contents

Quantitative determination of phenolics contents of the aqueous methanolic extracts of compound herbal formulation POL_4_ and its ingredients were carried out by an earlier method [40] with slight modification. Briefly, 1 ml of Folin-Ciocalteu reagent was added to the test material solution containing 1 mg in a volumetric flask and adjusted to 46 ml with distilled water. After 3 min, 3 ml of Na_2_CO_3_ (2%) was added to the mixture, which was afterward kept for 2 h at room temperature with continuous shaking on a shaker. The absorbance was measured at 760 nm by using spectrophotometer (Model DU-730, Beckman Instruments Inc, Palo Alto, CA, USA). Total phenolic content of the test materials were expressed as mg equivalent of gallic acid equivalent/g of the dried extract.

### Determination of total flavonoid contents

Amount of total flavonoid compounds of the aqueous methanolic extracts of POL_4_ and its ingredients were determined as described previously [41]. Around 1.5 ml of the test material solution was added to an equal volume of solution of 2% AlCl_3_.6H_2_O in methanol. The mixture was vigorously shaken and absorbance was recorded at 367 nm after 10 min of incubation. The absorption measured using a spectrophotometer (Model DU 730). Flavonoid contents were expressed as mg of quercetin equivalent/g of the extract.

### *In-vivo* blood pressure measurement in anaesthetized rats

SD rats (180–250 g) of either sex were used by following an earlier method [42]. The animals were randomly selected and were anesthetized with an intraperitoneal injection of thiopental sodium (purchased from Pharmacy of Aga Khan University Hospital, Karachi, Pakistan) at a dose of 40–70 mg/kg body weight. The arterial blood pressure was recorded by cannulating carotid artery filled with heparinized saline to a disposable blood pressure transducer (model MLT 0699) attached with bridge amplifier and a PowerLab data acquisition system (ADInstruments, Australia). Jugular vein was cannulated to inject different test material(s) to the animal. Before injecting 0.9% NaCl (0.1 ml/kg, saline) or equal volume of test substance(s) to rats, a period of about 10–15 min was given for stabilization. Heparin was purchased from Pharmacy of Aga Khan University Hospital, Karachi, Pakistan. Between each injected dose, the arterial pressure was allowed to return to normal or resting level. Prior to the assessment of the effect of the different doses (1–100 mg/kg) test material(s), standard drugs responses like acetylcholine (1 mg/kg) and norepinephrine (1 mg/kg) were given intravenously (jugular vein) to obtain the control responses. The difference between the steady state values before and after injection was recorded as the mean arterial pressure (MAP). MAP was calculated by using the formula PP = SP-DP where PP-pulse pressure, SP-systolic pressure and DP-diastolic pressure.

### Isolated rat aortic preparation

SD rats (180–250 g) of either sex were used randomly in this study and were anesthetized using Isoflurane (2–5% v/w) by inhalation in a closed chamber until achievement of deep anesthesia. Isoflurane was purchased from Pharmacy of Aga Khan University Hospital, Karachi, Pakistan. Dissection was carried out to isolate thoracic aortae and the aorta was cut into rings of around 2 to 3 mm. The rings were mounted in 5 ml water bath containing Kreb’s solution at 37 °C and carbogen [O_2_ (95%) and CO_2_ (5%)] was continuously supplied. The Kreb’s solution was prepared freshly on the day of experiment which consists of following ingredients in mM: NaCl 118.2, KCl 4.7, KH_2_PO_4_ 1.3, MgSO_4_ 1.2, NaHCO_3_ 25.0, Glucose 11.7 and CaCl_2_ 2.5 with pH 7.4. A pre-load of 2 g was provided as baseline tension and each tissue preparation was allowed to incubate for a period of 60 min. The changes in isometric tension were recorded and analyzed using the force transducer model (50–7905, Harvard Apparatus, USA), linked with a Trans-bridge model TBM4M and PowerLab data acquisition system (ML845, ADInstrument). The tissues were stabilized with phenylephrine (P.E 1 μM). The test materials (0.003 to 10 mg/ml) were tested for vasorelaxant potential against P.E (1 μM) and high K^+^ (80 mM)-induced contractions. For confirmation of calcium channel blocking (CCB) like activity, the Ca^++^ concentration-response curves (CRCs) were produced in Ca^++^-free medium in the absence and presence of test material(s) to observe if there is a non-parallel shift in the CRCs of Ca^++^ towards right with suppression of maximum response [15]. To determine the presence of vasoconstrictor constituents in test material(s), experiments were performed on resting baseline tone of the aortic preparations. First, tissues were stabilized with P.E until steady-state baseline tone; the vasoconstrictor effect of the test material(s) was presented as percent of P.E- induced contraction and has also been reproduced in the presence of phentolamine (α-adrenergic receptor antagonist) to identify its sensitivity with phentolamine.

### Isolated guinea-pig atria

Guinea-pigs of either sex were euthanized followed by anesthesia with Isoflurane. Dissection was carried out quickly and carefully to isolate right atria. The isolated atria was cleaned and mounted in 20 ml water bath containing Kreb’s solution maintained at 32 °C. The bathing solution was bubbled continuously with carbogen. The atria as applied 1 g resting tension and allowed to beat normally as it contains the pacemaker cells. Atrial tissue was allowed for an equilibrium period of around 30 min before application of any test material(s) or standard drug(s) like isoproterenol (1 μM) and acetylcholine (1 μM). A force-displacement transducer coupled to PowerLab data acquisition system (ADInstrument, Australia) was used to record the tension changes in the tissues [43].

### Data analysis

The experimental data presented as mean ± S.E.M (standard error of mean), n = number of experiments and EC_50_ (median effective concentration) values were reflecting as the geometric means with CI (confidence intervals) of 95%. Concentration response curves were constructed and analyzed by non-linear regression followed by sigmoidal curves. For determination of significant differences, one way ANOVA (analysis of variance) followed by Dunnet’s test or two-way ANOVA followed by Bonferroni’s post-test correction or unpaired *t*-test were applied. *P* values <0.05 were taken as significantly different. GrpahPad Prism (Version 4.00) software was used for constructing graphs and analysis.

## Results

### Phytochemical screening

Qualitative phytochemical screening of the aqueous-methanolic extract of  POL_4_ and its components have shown the presence of flavonoids, tannins, saponins, alkaloids, anthraquinones and coumarins.

### Total phenolic and flavonoid contents

Total phenolic and flavonoid contents of the aqueous-methanolic extract of POL_4_ and its ingredients have been found in varying proportion. Phenolic contents were found in highest proportion in *G. sylvestre* followed by Ci.Cr > POL_4_.Cr > Ns.Cr > Tfg.Cr (Table 1). While flavonoids were found highest in *T. foenum graecum* followed by Gs.Cr > POL_4_.Cr > Ci.Cr > Ns.Cr (Table 1).

**Table 1:** Total phenolic and flavonoid contents of POL_4_ and its ingredients

Test materials	Total phenolic contents (mg GAE/g of extract)	Total flavonoid contents (mg QE/g of extract)
POL_4_.Cr	18.56 ± 1.0	66.68 ± 0.87
Ns.Cr	11.27 ± 0.76	17.19 ± 1.52
Ci.Cr	24.73 ± 0.20	64.86 ± 0.86
Tfg.Cr	10.15 ± 0.64	87.85 ± 0.50
Gs.Cr	39.88 ± 0.78	75.64 ± 0.70

Results represent mean ± SD of 3 measurements

*Abbreviations*: *POL*_*4*_ *.Cr* the crude extract of polyherbal formulation, *Ci.Cr* the crude extract of *C. intybus*, *Gs.Cr* the crude extract of *G. sylvestre*, *Ns.Cr* the crude extract of *N. sativa*, *Tfg.Cr* the crude extract of *T. foenum graecum*, *GAE* gallic acid equivalent, *QE* quercetin equivalent

### Effect on blood pressure in anaesthetized rats

**Fig. 1:**
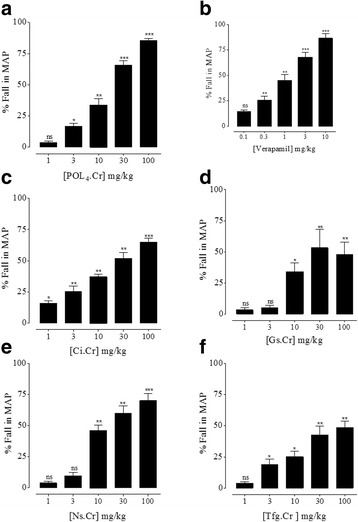
Blood pressure lowering effect of (**a**) the crude extract of polyherbal formulation (​POL_4_.Cr), (**b**) verapamil, (**c**) the crude extracts of *C. intybus *(Ci.Cr), (**d**) *G. sylvestre* (Gs.Cr), (**e**) *N. sativa *(Ns.Cr) and (**f**) *T. foenum graecum* (Tfg.Cr) in normotensive anesthetized rats. The data depicts mean ± SEM of 3 to 5 individual experiments using 7 to 10 animals. ns, non-significant, *p < 0.05, **p < 0.01 and ***p < 0.001vs saline (0.1 ml/kg) used as control (One-way ANOVA followed by Dunnett’s test)

In normotensive anaesthetized SD rats, intravenous administration of polyherbal formulation (POL_4_) and its ingredients (*C. intybus, G. sylvestre, N. sativa* and *T. foenum graecum*) caused a dose-dependent (1, 3, 10, 30 and 100 mg/kg) fall in MAP (Fig. 1). Verapamil has also decreased MAP dose-dependently (0.1–10 mg/kg) as seen in Fig. 1. The % maximum effects (E_max_) of the parent formulation, its ingredients and verapamil at respective tested concentrations have been listed together in Table 2. The parent formulation has been found the most effective followed by Ns.Cr ≅ Ci.Cr > Tfg.Cr > Gs.Cr.

**Table 2:** Percentage maximum drop in blood pressure (%E_max_) by the test materials in normotensive anesthetized rats

Test materials	Concentration	E_max_
POL_4_.Cr	100 (mg/ml)	85.33 ± 1.76 (*n* = 4)
Verapamil	10 (μM)	86.66 ± 4.41^ns^ (*n* = 4)
Ci.Cr	100 (mg/ml)	65.00 ± 2.88 **(*n* = 3)
Gs.Cr	30 (mg/ml)	53.33 ± 14.53**(*n* = 5)
Ns.Cr	100 (mg/ml)	70.00 ± 5.77 **(*n* = 5)
Tfg.Cr	100 (mg/ml)	48.5 ± 5.00**(*n* = 4)

Data represents mean ± SEM of 3 to 5 experiments. ns, non-significant, **p < 0.01 vs POL_4_.Cr (One-way ANOVA followed by Dunnett’s test)

*Abbreviations*: *POL*_*4*_ *.Cr* the crude extract of poly herbal formulation, *Ci.Cr* the crude extract of *C. intybus*, *Gs.Cr* the crude extract of *G. sylvestre*, *Ns.Cr* the crude extract of *N. sativa*, *Tfg.Cr* the crude extract of *T. foenum graecum*

### Effect on isolated rat aortic preparation

**Fig. 2:**
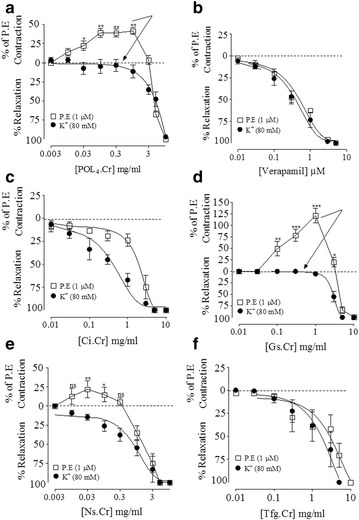
Vasomodulatory effect of (**a**) the crude extract of polyherbal formulation (​POL4.Cr), (**b**) verapamil, (**c**) the crude extracts of *C. intybus* (Ci.Cr), (**d**) *G. sylvestre* (Gs.Cr), (**e**) *N. sativa* (Ns.Cr) and (**f**) *T. foenum graecum *(Tfg.Cr) against phenylephrine and K^+^ (80 mM)-induced contractions in rat aortae. The data represent mean ± SEM of 4 to 7 experiments using tissues of 5 to 6 animals. ns, non-significant, *p < 0.05, **p < 0.01 and ***p < 0.001vs. baseline (Unpaired *t*-test)

**Fig. 3:**
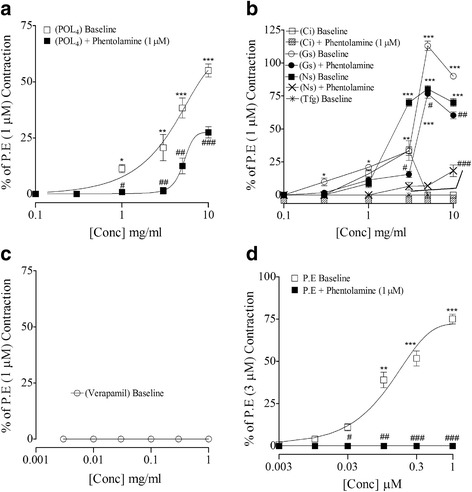
Vasoconstrictor effect of (**a**) ​POL_4_.Cr, (**b**) its ingredients, (**c**) verapamil and (**d**) phenylephrine (P.E) without and with phentolamine on basal tone of rat aortae. The data show mean ± SEM of 4 to 8 experiments using tissues of 4 to 5 animals.*p < 0.05, **p < 0.01 and***p < 0.001 vs. baseline effects, while ^**#**^ *p* < 0.05, ^**##**^ *p* < 0.01 and ^**###**^ *p* < 0.001 vs. vasoconstriction in the presence of phentolamine (Unpaired *t*-test). Abbreviations: POL_4_.Cr, the crude extract of poly-herbal formulation; Ci, the crude extract of *C. intybus*; Gs, the crude extract of *G. sylvestre*; Ns, the crude extract of *N. sativa*; Tfg, the crude extract of *T. foenum graecum*

The vasomodulatory effect of parent polyherbal formulation (POL_4_) and its ingredients (*C. intybus, G. sylvestre*, *N. sativa* and *T. foenum graecum*) were tested against high K^+^ (80 mM) and P.E (1 μM)-induced contractions, while to determine the presence of vasospastic activity, test materials were challenged on stabilized basal tone of isolated rat aortic preparations. Against P.E-induced contractions, POL_4_, *G. sylvestre* and *N. sativa* have shown dual activities, initially vasoconstriction at 0.01–1 mg/ml followed by relaxation at 3–10 mg/ml. However, *C. intybus* and *T. foenum graecum* caused complete relaxation of P.E-induced contractions at 0.03–10 mg/ml, similar to the activity pattern of verapamil (0.03–3 μM) as seen in Fig. 2. The summary of vasomodulatory effects of POL_4_ and its ingredients has been shared in upper panel of Table 3. Against high K^+^-induced contractions, the parent formulation and its ingredients caused inhibition at 0.01–10 mg/ml, similar to the effect of verapamil at 0.03–3 μM as shown in Fig. 2. Their respective EC_50_ values are presented in Table 3. When tested on basal/resting tone of rat aorta, except *T. foenum graecum* which showed activity pattern as of verapamil, the parent formulation and its constituents exhibited vasoconstriction at higher concentrations similar to the effect of phenylephrine. When reproduced in tissues pretreated with phentolamine (1 μM), the observed vasocontractile effects were found attenuated as seen in Fig. 3. The % age maximum vasoconstriction (V_max_) of POL_4_ and its elements in the absence and presence of phentolamine has been listed in Table 4.

**Table 3:** Percentage maximum vasoconstriction (%V_max_) and EC_50_ values of the test materials against P. E or K^+^ (80 mM)-induced contractions in rat aortic preparations

Parameters	Test materials					
	POL_4_ .Cr	Verapamil	Ci. Cr	Gs. Cr	Ns. Cr	Tfg. Cr
Effect against P.E (1 μM)-induced contractions						
% V_max_/Conc. (mg/ml)	141.43 ± 4.63/1	-	-	221.66 ± 15.89***/1	131.95 ± 6.95*/0.03	-
Complete inhibition with resultant EC_50_ values with 95% confidence interval	-	0.417 mg/ml (0.31–0.54)	2.01 mg/ml (1.62–2.49)	-	-	2.29 mg/ml (1.15–4.56)
Effect against K^+^ (80 mM)-induced contractions						
Complete inhibition with resultant EC_50_ values	4.70 mg/ml (3.94–5.60)	0.27 mg/ml (0.18–0.39)	0.31 mg/ml (0.19–0.52)	3.13 mg/ml (2.15–4.56)	0.68 mg/ml (0.41–1.14)	1.40 mg/ml (0.91–2.15)

Data represents mean ± SEM of 4 to 7 experiments

*Abbreviations: POL*_*4*_ *.Cr* the crude extract of poly herbal formulation, *Ci.Cr* the crude extract of *C. intybus*, *Gs.Cr* the crude extract of *G. sylvestre*, *Ns.Cr* the crude extract of *N. sativa*, *Tfg.Cr* the crude extract of *T. foenum graecum*

*p < 0.05 and ***p < 0.001 vs. POL_4_.Cr (Unpaired *t*-test)

**Table 4:** Percentage maximum vasoconstriction (%V_max_) of test materials without and with phentolamine in rat aortic tissues

Test materials	Concentration producing V_max_	%V_max_ without phentolamine	%V_max_ with phentolamine (1 μM)
POL_4_.Cr	10 (mg/ml)	55 ± 2.88	27.5 ± 2.50^###^
Ci.Cr	3 (mg/ml)	33.66 ± 7.53**	Blocked^###^
Gs.Cr	3 (mg/ml)	113 ± 3.51***	76.66 ± 3.33^###^
Ns.Cr	5 (mg/ml)	80.33 ± 2.60**	18.33 ± 4.41^###^
Tfg.Cr	Did not exhibited any vasoconstriction		
Verapamil	Did not exhibited any vasoconstriction		
P.E	1 (μM)	75.19 ± 2.16 ***	Blocked^###^

Data represents mean ± SEM of 4 to 8 experiments

*Abbreviations: POL*_*4*_ *.Cr* the crude extract of poly herbal formulation, *Ci.Cr* the crude extract of *C. intybus*, *Gs.Cr* the crude extract of *G. sylvestre*, *Ns.Cr* the crude extract of *N. sativa*, *Tfg.Cr* the crude extract of *T. foenum graecum*, *P.E* Phenylephrine

ns, non-significant, **p < 0.01 and ***/^**###**^ *p* < 0.001

* shows a comparison of %V_max _of test materials without phentolamine vs. POL_4_.Cr (One-way ANOVA followed by Dunnett’s test), while ^**#**^ shows a comparison between %V_max_ values without phentolamine vs. respective %V_max _values with phentolamine (Unpaired *t*-test)

**Fig. 4:**
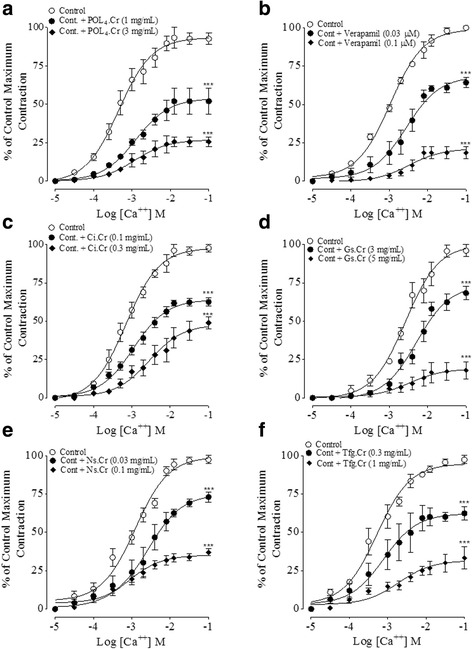
Concentration-response curves of Ca^++^ constructed in absence and presence of (**a**) the crude extract of polyherbal formulation (​POL4.Cr), (**b**) verapamil, (**c**) the crude extracts of *C. intybus *(Ci.Cr), (**d**) *G. sylvestre* (Gs.Cr), (**e**) *N. sativa *(Ns.Cr) and (**f**) *T. foenum graecum *(Tfg.Cr) in rat aortae. The data denote mean ± SEM of 4 to 7 experiments using tissues of 4 to 6 animals ***p < 0.001 vs. Control CRCs of Ca^++^ (Two-way analysis of variance (ANOVA) followed by Bonferroni’s post-test correction)

To confirm the Ca^++^ antagonist like activity, when the CRCs of Ca^++^ were constructed in the absence and presence of increasing concentrations of POL_4_ and its components, these produced a non-parallel rightward shift in the concentration-related curves of Ca^++^ with suppression of maximum effect at concentrations range of 0.03–5 mg/ml, similar to verapamil (0.03 and 0.1 μM) as seen in Fig. 4. The maximum contractions of Ca^++^ at highest concentration in the absence and presence of respective test material(s) is presented in Table 5.

**Table 5:** Percentage maximal response of Ca^++^ (% E_max_) without and with test materials in rat aortic tissues

Test materials	Concentration	% E_max_ of Ca^++^ ± SEM, *n* = 4–7	*P* value
Control	-	100 ± 0	-
POL_4_.Cr (mg/ml)	1	52.66 ± 8.62	*p* < 0.001
	3	25.66 ± 3.18	*p* < 0.001
Verapamil (μM)	0.03	63.85 ± 4.00	*p* < 0.001
	0.1	18.65 ± 4.09	*p* < 0.001
Ci.Cr (mg/ml)	0.1	62.33 ± 3.84	*p* < 0.001
	0.3	49.00 ± 4.00	*p* < 0.001
Gs.Cr (mg/ml)	3	68.33 ± 4.41	*p* < 0.001
	5	17.93 ± 5.50	*p* < 0.001
Ns.Cr (mg/ml)	0.03	73.00 ± 3.39	*p* < 0.001
	0.1	37.00 ± 2.38	*p* < 0.001
Tfg.Cr (mg/ml)	0.3	62.33 ± 4.33	*p* < 0.001
	1	33.33 ± 7.26	*p* < 0.001

*p*-value were obtained by comparing the concentration–response curves of Ca^++^ in the absence (control) and presence of respective test material(s) using two-way analysis of variance (ANOVA) followed by Bonferroni’s post-test correction

*Abbreviations: POL*_*4*_ *.Cr* the crude extract of poly herbal formulation, *Ci.Cr* the crude extract of *C. intybus*, *Gs.Cr* the crude extract of *G. sylvestre*, *Ns.Cr* the crude extract of *N. sativa*, *Tfg.Cr* the crude extract of *T. foenum graecum*

### Effect on isolated guinea-pig atria

**Fig. 5:**
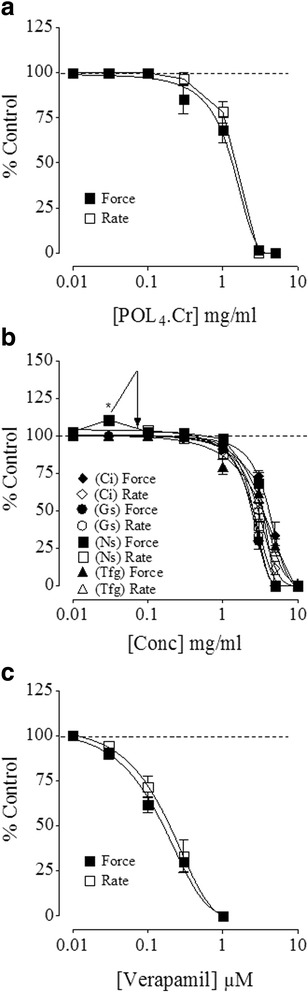
Modulatory effect of (**a**) ​POL_4_.Cr, (**b**) its ingredients and (**c**) verapamil  on force and rate of contraction in isolated guinea-pig atria. Data represent mean ± SEM of 3 to 7 individual experiments using tissues of 4 to 6 animals. *p < 0.05vs control (Unpaired *t*-test). Abbreviations: POL_4_.Cr, the crude extract of polyherbal formulation; Ci, the crude extract of *C. intybus*; Gs, the crude extract of *G. sylvestre*; Ns, the crude extract of *N. sativa*; Tfg, the crude extract of *T. foenum graecum*

In isolated guinea-pig atria, the parent formulation and three of its constituents (*C. intybus, G. sylvestre* and *T. foenum graecum*) completely depressed the force and rate of spontaneously beating atria at concentrations range (0.1–10 mg/ml) as shown in Fig. 5a and b, similar to verapamil which also inhibited the force and rate of atrial contraction at 0.01 to 1 μM (Fig. 5c). However, *N. sativa* initially showed mild cardiac stimulation in terms of an increase in the force of contraction followed by inhibition, while no accelerating effect was observed on the rate of contraction of atria as seen in Fig. 5b. Table 6 shows respective EC_50_ values of parent formulation and its ingredients against force and rate of contractions of the isolated atrial tissues.

**Table 6:** EC_50_ values of the test materials against force and rate of contractions in guinea-pig atria

Test materials	Force (inhibition) with resultant EC_50_ values with 95% confidence interval/Mean ± SEM, *n* = 3–7	Rate (inhibition) with resultant EC_50_values, *n* = 3–7
POL_4._Cr	1.28 mg/ml (1.08–1.52)	1.22 mg/ml (0.70–2.15)
Verapamil	0.14 mg/ml (0.11–0.83)	0.19 mg/ml (0.15–0.24)
Ci.Cr	4.18 mg/ml (3.92–4.46)	3.16 mg/ml (2.95–3.39)
Gs.Cr	2.47 mg/ml (2.24–2.72)	3.16 mg/ml (3.03–3.30)
Ns.Cr	Mild cardiac stimulation (110.66 ± 2.33%) at 0.03 mg/ml followed by inhibition at 1–10 mg/ml.	2.47 mg/ml (2.06–2.95)
Tfg.Cr	3.65 mg/ml (3.21–4.16)	3.516 mg/ml (3.23–3.82)

*Abbreviations: POL*_*4*_ *.Cr* the crude extract of poly herbal formulation, *Ci.Cr* the crude extract of *C. intybus*, *Gs.Cr* the crude extract of *G. sylvestre*, *Ns.Cr* the crude extract of *N. sativa*, *Tfg.Cr* the crude extract of *T. foenum graecum*

## Discussion

On account of the medicinal use of POL_4_ and its the ingredients in cardiometabolic disorders like diabetes and associated hypertension [17], when tested in anesthetized rats, POL_4_ and its ingredients attenuated the mean arterial pressure (MAP) in a dose-dependent manner. Interestingly, the parent formulation was found the most effective followed by Ns.Cr ≅ Ci.Cr > Tfg.Cr > Gs.Cr. Our findings on the part of *N. sativa* are in line with earlier report on its antihypertensive efficacy [32]. However, this study is reporting for the first time, the blood pressure lowering efficacy of *C. intybus* and *G. sylvestre* in normotensive rats. These findings are also providing a rationale to their medicinal use as cardiotonic or hypotensive [17, 19]. The blood pressure lowering effects are also in agreement with the prior findings related to vasorelaxant mechanisms on the part of *C. intybus* [27] and *T. foenum graecum* [30, 31], which might be playing a pivotal role in overall effectiveness of these plants in hypertension. On the contrary, Preuss et al. [44] reported that *G. sylvestre* did not show antihypertensive effect in spontaneously hypertensive rats, however, we did find its efficacy but with varying pattern in normotensive rats which needs further investigations using multiple models and species to attest or oppose its therapeutic utility in hypertension.

Blood pressure is the product of cardiac output and peripheral resistance [45]. An increase in any of these two determinants can develop hypertension. Antihypertensive drugs target both components either alone or in combination. The preferred approach would be to cause a predominantly reduction in peripheral vascular resistance along with cardiac output.

To explore the effect of parent formulation and its components on vascular resistance, in pre-contracted rat aortic preparations, POL_4_ and its ingredients caused inhibition of K^+^ (80 mM)-induced contractions, where Ci.Cr was found the most potent followed by Ns.Cr > Tfg.Cr > Gs.Cr ≅ POL_4_. Based on inhibitory effects of test material(s) against K^+^ (80 mM)-induced contractions, to further attest the presence of Ca^++^ antagonist like activity on the part of test material(s), the concentration-response curves (CRCs) of Ca^++^ were constructed in the absence and presence of different concentrations of POL_4_ and its ingredients, respectively. All produced a non-parallel rightward shift in CRCs of Ca^++^ with suppression of the maximum effect, similar to activity pattern of verapamil, a known Ca^++^ antagonist [46]. Against phenylephrine (P.E)- induced contraction, *C. intybus* and *T. foenum graecum* showed complete relaxation, however, POL_4_, *G. sylvestre* and *N. sativa* showed initially vasoconstriction followed by relaxation at higher tested concentrations. The inhibitory potential of POL_4_ and its ingredients against K^+^ (80 mM) and P.E signify their ability to interfere with Ca^++^ entry through voltage-dependent channels (VDCs) and receptor-operated channels (ROCs). This is evident as K^+^ (80 mM) and P.E (1 μM) mediate contraction by influx of extracellular Ca^++^ through VDCs and ROCs, respectively, resulting in increased intracellular Ca^++^ levels trigging sustained contractions [43, 47]. Based on observed (vasoconstrictor and/or vasodilator) effects of test material(s) on P.E-induced contractions, the parent formulation and its ingredients were studied for the presence of vaso-stimulant activity on the baseline status of isolated rat aortic preparations. All exhibited vasoconstriction with varying efficacy, being Gs.Cr the most potent followed by Ns.Cr > POL_4_ > Ci.Cr), similar to the effect of phenylephrine. However, *T. foenum graecum* was devoid of any vasoconstriction, similar to the effect of verapamil. When these effects were studied in the presence of phentolamine, an α- adrenergic antagonist, all showed partial sensitivity to phentolamine except *C. intybus* which was found completely phentolamine-sensitive. These data suggest the presence of multiple types of vasoconstrictor constituents like phentolamine sensitive and insensitive, which needs further studies to characterize. It was interesting to note that despite the presence of vasoconstrictor constituents both in parent formulation and most of its ingredients, these did not increase blood pressure in anaesthetized rats, which might be because of the dominant role of endogenous mediators in intact body systems offering blockade to vasoconstrictor constituents. On the other hand, the co-existence of strong vasorelaxant and cardio depressant elements in the parent formulation and its ingredients may not be allowing the expression of vasoconstrictor elements to dominate and cause hypertension in the in vivo system.

To determine cardio-suppressant activity contributing in attenuating cardiac output, when the parent formulation and its ingredients were tested on spontaneously beating atrial preparations of guinea-pigs, POL_4_ and three of its ingredients showed exclusively negative inotropic and chronotropic effects similar to the effect of verapamil. However, *N. sativa* showed mild cardiac stimulation in terms of an increase in the force of contraction, which is also in support to its medicinal use as cardiac stimulant in the form of a formulation (Lubub-Al-Asrar) [16]. The probable mechanism likely to be involved in cardio-depressant activities seems Ca^++^ antagonist pathway, while mild cardiac stimulant effect on the part of *N. sativa* needs further exploration to confirm. The standard Ca^++^ antagonist like verapamil which is known to lower blood pressure [48, 49] partly by causing cardiac suppression via inhibition of inward slow current at the time of action potential [50]. The observed cardiac inhibitory effect of the test material(s) might be attenuating cardiac output leading to a decrease in the blood pressure. Our findings are in line with the cardiac inhibitory effect of medicinal plants causing reduction in the cardiac output, thus resulting in decreased blood pressure [15, 43, 51–53]. In summary, it appears that nature has composed the opposing mechanisms not allowing the vasodilator effect to cross the desired limit thus overriding excessive drop in blood pressure, which has usually been observed when using standard antihypertensive drugs [45]. The co-existence of components with combating activities have been found common in many plants like *Viola odorata* [43], *Carthamus oxycantha* [47], *Curcuma long* [54], *Acorus calamus* [55] and piperine, an alkaloid of *Piper longum* and *Piper nigrum* [56] and is also in agreement with the concept that natural products in their original form possess synergistic and/or side effects nullifying combinations [11]. Varied distribution of total phenolic and flavonoid in POL_4_ and its constituents is also providing strength to their cardiovascular benefits as phenolic and flavonoids are known for their effectiveness in cardiovascular disorders [50, 57, 58]. On the other hand, these findings suggest that excluding *G. sylvestre* from POL_4_ formulation may result in a better product with improved efficacy in treating hypertension.

## Conclusions

This study indicates that POL_4_ and its ingredients possess blood pressure lowering effect predominantly mediated through inhibition of Ca^++^ influx via membranous Ca^++^ channels and receptor (α-adrenergic) operated pathways. The additional presence of vasoconstrictor agents (partially phentolamine-sensitive) in parent formulation possibly because of *G. sylvestre* and *N. sativa* may be meant to suppress the excessive hypotension, a known adverse effect associated with the use of antihypertensive drugs when used at higher doses and/or for longer duration. Thus, this study provides a rationale to the medicinal use of POL_4_ and its ingredients in cardiovascular disorders like hypertension.

## Abbreviations

ACh: Acetylcholine
CCB: Ca^++^ channel blockade
CI: Confidence interval
Ci.Cr: Aqueous methanolic extract of *Cichorium intybus*
CRCs: Concentration-response curves
EC_50_: Effective concentration producing 50% effect
Gs.Cr: Aqueous methanolic extract of *Gymnema sylvestre*
High K^+^: K^+^ (80 mM)
i.p: intraperitoneally
MAP: Mean arterial pressure
Ns.Cr: Aqueous methanolic extract of *Nigella sativa*
POL_4_.Cr: Aqueous methanolic extract of polyherbal formulation
ROCs: Receptor-operated channels
Tfg.Cr: Aqueous methanolic extract of *Trigonella foenum graecum*
VDCs: Voltage-dependent channels
